# Editorial: Host-microbiota interactions in IBD: immune modulation and barrier function

**DOI:** 10.3389/fcimb.2026.1869268

**Published:** 2026-06-03

**Authors:** Hongcai Li, Sagnik Nag, Gratiela Gradisteanu Pircalabioru, Ralf Weiskirchen

**Affiliations:** 1 College of Food Science and Engineering, Northwest A&F University, Yangling, Shaanxi, China; 2 Jeffrey Cheah School of Medicine and Health Sciences, Monash University Malaysia, Jalan Lagoon Selatan, Bandar Sunway, Selangor, Malaysia; 3 Department of Botany and Microbiology, Faculty of Biology, University of Bucharest, Bucharest, Romania; 4 eBio-hub Centre of Excellence in Bioengineering, National University for Science and Technology, Politehnica Bucharest, Bucharest, Romania; 5 Academy of Romanian Scientists, Bucharest, Romania; 6 Institute of Molecular Pathobiochemistry, Experimental Gene Therapy and Clinical Chemistry (IFMPEGKC), RWTH University Hospital Aachen, Aachen, Germany

**Keywords:** gut microbiota, host-microbiota, IBD, metabolic diseases, potential associations

## Introduction

Inflammatory bowel diseases (IBD), including Crohn’s disease and ulcerative colitis, are chronic inflammatory disorders of the gastrointestinal tract characterized by a multifactorial etiology involving genetic susceptibility, environmental influences, immune dysregulation, and alterations of the intestinal microbiota (Ning et al., 2024). Over the past decades, growing evidence has highlighted the central role of host-microbiota interactions in maintaining intestinal homeostasis. Under physiological conditions, the gut microbiota contributes to metabolic functions, supports epithelial barrier integrity, and shapes mucosal immune responses (Paudel et al., 2024). In IBD, however, this balanced relationship becomes disrupted, leading to microbial dysbiosis, impaired barrier function, and aberrant immune activation that together sustain chronic intestinal inflammation.

**Figure 1:**
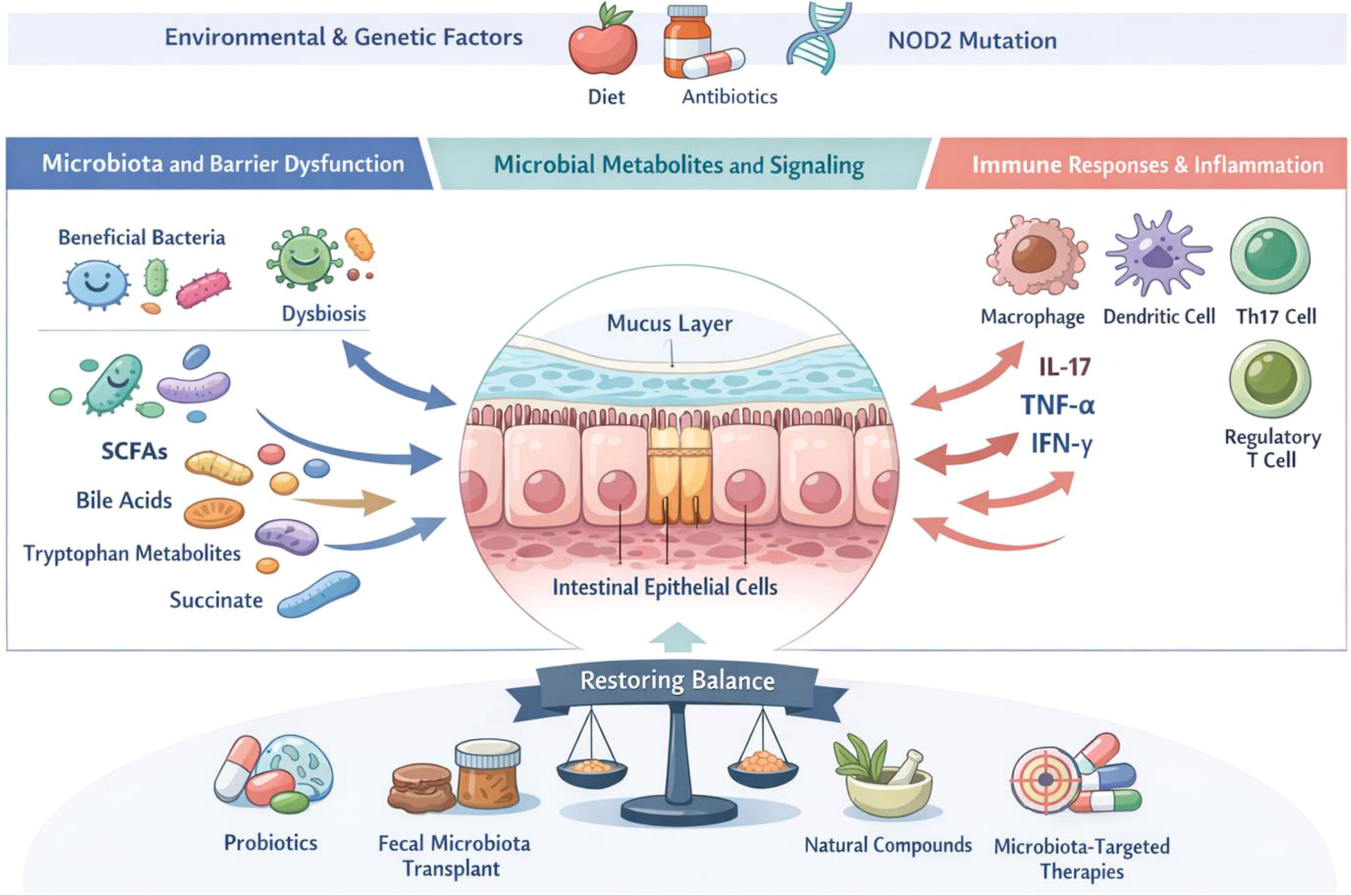
Conceptual overview of host-microbiota interactions influencing immune regulation and intestinal barrier function in inflammatory bowel disease (IBD). In normal conditions, the gut microbiota plays a key role in maintaining intestinal balance by producing metabolites such as short-chain fatty acids, bile acids, and tryptophan derivatives. These metabolites support the integrity of the epithelial barrier and regulate immune responses in the mucus layer. The intestinal barrier, consisting of epithelial cells, tight junction proteins, mucus layers, and antimicrobial peptides, is crucial in controlling communication between luminal microorganisms and host immune cells. In IBD, microbial dysbiosis and environmental factors like antibiotic exposure or genetic susceptibility disrupt this balance. Changes in microbial communities and metabolites lead to pro-inflammatory signaling, macrophage activation, and T helper cell polarization, while also impairing regulatory pathways. Simultaneously, dysfunction in the epithelial barrier allows for microbial translocation, further exacerbating inflammation and perpetuating a cycle of immune activation and tissue damage. New therapeutic approaches such as microbiota-targeted interventions, fecal microbiota transplantation, natural compounds, and metabolite-based therapies, aim to restore microbial balance, strengthen barrier integrity, and re-establish immune homeostasis. This conceptual framework highlights the interconnected pathways explored in the studies included in this Research Topic.

The intestinal barrier represents a critical interface between the host and the luminal microbial environment (Zhang et al., 2024). It consists of epithelial cells, tight junctions, mucus layers, antimicrobial peptides, and immune components that together regulate microbial containment while allowing beneficial host-microbe communication. Disturbances in this barrier system can permit increased microbial translocation and the activation of pro-inflammatory pathways. Conversely, inflammatory signals further compromise epithelial integrity, establishing a self-perpetuating cycle of barrier dysfunction and immune dysregulation (**Figure 1**). Understanding how microbial communities interact with immune and epithelial compartments is therefore essential for elucidating the mechanisms underlying IBD pathogenesis.

The Research Topic “*Host-Microbiota Interactions in IBD: Immune Modulation and Barrier Function*” brings together fourteen articles that explore different dimensions of this complex interplay. The contributions span mechanistic studies, experimental models, epidemiological analyses, and comprehensive reviews that collectively highlight how microbial communities and their metabolites influence immune signaling, barrier integrity, and disease progression. In the following sections, these contributions are organized into three thematic areas reflecting major research directions within this field.

## Microbiota-driven immune modulation and barrier dysfunction

Several contributions in this Research Topic focus on how microbial dysbiosis influences immune responses and intestinal barrier integrity. Tian et al. investigated the role of gut microbiota in the progression of experimental autoimmune myasthenia gravis (EAMG) using a Lewis rat model. The study demonstrated that disease development is accompanied by pronounced microbial dysbiosis, increased levels of pro-inflammatory cytokines including IL-17, IFN-γ, and TNF-α, and reduced anti-inflammatory IL-10. These alterations were associated with elevated circulating lipopolysaccharide levels and decreased expression of barrier proteins such as claudin-1 and MUC2, indicating impaired intestinal permeability. Importantly, fecal microbiota transplantation from diseased animals induced inflammatory responses and barrier disruption in recipient rats, suggesting a causal role of dysbiotic microbiota in disease progression.

Experimental evidence linking microbiota alterations to inflammatory responses is further supported by studies examining therapeutic interventions in colitis models. Yin et al. evaluated the effects of hyperoside, a plant-derived flavonoid, in a dextran sulfate sodium (DSS)-induced mouse model of ulcerative colitis. Hyperoside treatment significantly reduced disease severity, suppressed pro-inflammatory cytokine production, restored intestinal barrier proteins such as MUC2 and ZO-1, and alleviated anxiety-like behaviors associated with intestinal inflammation. Microbiome analysis revealed substantial reshaping of the gut microbial community, and antibiotic depletion experiments demonstrated that the beneficial effects of hyperoside depend on the presence of gut microbiota.

Similarly, Fu et al. investigated the protective effects of *Taraxacum* extract in DSS-induced colitis. Treatment improved clinical and histological indicators of colitis, reduced inflammatory cytokine levels, and enhanced antioxidant capacity. At the same time, the extract restored the expression of tight junction proteins including claudin-1, occludin, and ZO-1 and promoted beneficial shifts in gut microbiota composition accompanied by increased short-chain fatty acid production. Together, these studies highlight how modulation of microbial communities and barrier integrity can mitigate intestinal inflammation.

The importance of microbial composition for therapeutic success is also emphasized by Dai Z. et al., who examined the influence of donor microbiota profiles on fecal microbiota transplantation outcomes. By stratifying donors based on the abundance of health-associated bacterial taxa, the authors demonstrated that microbiota derived from donors enriched in beneficial genera produced stronger therapeutic effects in experimental colitis models. These findings underscore the importance of donor microbial characteristics in determining the efficacy of microbiota-based interventions for IBD.

## Microbial metabolites and host signaling pathways

A second group of contributions addresses the role of microbial metabolites and host signaling pathways in shaping intestinal inflammation. Dai M. et al. reviewed the emerging role of succinate metabolism in IBD pathogenesis. Succinate, an intermediate of the tricarboxylic acid cycle that can also be produced by intestinal bacteria, accumulates under conditions of dysbiosis and metabolic stress. Elevated succinate levels activate the SUCNR1 receptor and stabilize HIF-1α signaling, promoting macrophage M1 polarization and Th17 differentiation while suppressing regulatory T cell responses. Through these mechanisms, succinate functions as both a metabolic intermediate and an immunological signaling molecule linking microbial metabolism to intestinal inflammation.

The broader role of microbial metabolites in intestinal homeostasis is further discussed in the review by Shen et al., which summarizes how short-chain fatty acids, bile acids, and tryptophan derivatives regulate epithelial barrier integrity and immune responses. These metabolites interact with host receptors such as G-protein-coupled receptors, the farnesoid X receptor, and the aryl hydrocarbon receptor to modulate inflammatory signaling pathways. Alterations in microbial metabolism and reduced availability of these protective metabolites are increasingly recognized as key contributors to disease progression in IBD.

Complementing these perspectives, Hu et al. propose the antimicrobial peptide-antibiotic-microbiota triad as a conceptual framework explaining disrupted antimicrobial defense in IBD. Antimicrobial peptides produced by epithelial and immune cells normally shape microbial communities and support mucosal barrier function. Genetic susceptibility factors, including NOD2 mutations, together with environmental influences such as antibiotic exposure, can disturb this balance, leading to dysbiosis and chronic inflammation. The authors discuss therapeutic approaches aimed at restoring antimicrobial homeostasis, including strategies that enhance antimicrobial peptide activity or selectively modulate microbial communities.

A related perspective is provided by Giju et al., who review the microbial-metabolic axis in IBD and discuss emerging precision-medicine strategies. By integrating advances in metagenomics, metabolomics, and computational modeling, the authors highlight how microbial metabolic signatures may serve as predictive biomarkers for disease activity and therapeutic responses. These approaches could support personalized interventions targeting microbial pathways involved in intestinal inflammation.

## Microbiota in disease progression and clinical contexts

Beyond mechanistic insights, several contributions explore the broader clinical and epidemiological implications of microbiota alterations. Lyu et al. review the bidirectional interactions between gut microbiota and macrophages in the development of colitis-associated colorectal cancer (CAC), a severe long-term complication of IBD. Pathogenic bacteria such as *Fusobacterium nucleatum* and *Escherichia coli* can promote inflammatory signaling and macrophage polarization, whereas beneficial microbes including *Bifidobacterium* and *Lactobacillus* support immune regulation and barrier protection. The review highlights how microbial metabolites influence macrophage signaling pathways and discusses therapeutic strategies targeting this microbiota-macrophage axis to prevent inflammation-driven tumorigenesis.

Zheng et al. expand the perspective beyond intestinal inflammation by examining the interplay between gastrointestinal dysfunction and microbiota alterations in sepsis. During sepsis, intestinal barrier disruption and immune dysregulation lead to profound microbial imbalance characterized by reduced diversity and expansion of opportunistic pathogens. These microbial changes disrupt metabolic pathways and contribute to systemic inflammation and multi-organ dysfunction through gut–organ axes, including the gut-liver, gut-lung, and gut-brain pathways. The authors discuss potential microbiome-based interventions aimed at restoring intestinal homeostasis in critically ill patients.

Age-related differences in microbial communities are explored by Toto et al., who analyzed bacterial and fungal metagenomes in pediatric and adult patients with ulcerative colitis. While bacterial communities showed relatively similar compositions across age groups, fungal communities differed markedly between children and adults. Pediatric patients exhibited enrichment of inflammation-associated fungal taxa and reduced abundance of health-associated species, suggesting that life stage may influence host-microbiota interactions and immune responses in ulcerative colitis.

At the population level, Li X. et al. examined global trends in IBD burden from 1990 to 2023 in relation to microbiota dysbiosis. By integrating epidemiological data from the Global Burden of Disease study with microbiome sequencing datasets, the authors observed that regions with more pronounced microbial imbalance experienced faster increases in IBD incidence and disability-adjusted life years. Declines in beneficial bacteria and reduced microbial diversity were associated with greater disease severity, supporting the concept that microbial ecosystem changes may contribute to the rising global burden of IBD.

Finally, two reviews provide broader clinical perspectives on the role of microbiota in ulcerative colitis and IBD management. Chen and Liu summarize current understanding of ulcerative colitis pathogenesis, emphasizing the contributions of microbial dysbiosis, immune dysregulation, and barrier dysfunction. Li Y. further reviews how microbiota alterations influence immune responses and intestinal homeostasis in IBD and discusses microbiota-targeted therapeutic strategies, including probiotics, dietary interventions, and fecal microbiota transplantation. Although these approaches show promise, the authors emphasize the need for further research to optimize their clinical application.

## Concluding remarks

Together, the contributions assembled in this Research Topic illustrate the multifaceted nature of host-microbiota interactions in inflammatory bowel disease. From mechanistic studies of immune and epithelial signaling to investigations of microbial metabolites, therapeutic interventions, and population-level trends, the collected articles highlight how disturbances in the dialogue between host and microbiota can influence disease onset, progression, and treatment outcomes.

A common theme emerging from these studies is the recognition that intestinal inflammation cannot be fully understood without considering the complex ecological and metabolic interactions occurring within the gut microbiome. Advances in microbiome research, systems biology, and translational medicine are increasingly enabling the identification of microbial and metabolic pathways that may serve as diagnostic markers or therapeutic targets.

By bringing together diverse perspectives from microbiology, immunology, and clinical research, this collection contributes to a more integrated understanding of intestinal homeostasis and its disruption in IBD. Continued exploration of host-microbiota interactions will be essential for the development of innovative therapeutic strategies aimed at restoring microbial balance, strengthening barrier function, and ultimately improving outcomes for patients with inflammatory bowel diseases.
